# Supplementary material for: Deep learning and direct sequencing of labeled RNA captures transcriptome dynamics
Source: bioRxiv. 2023 Nov 17:2023.11.17.567581. Preprint. [Version 1] doi: 10.1101/2023.11.17.567581 (PMC10680836; doi:10.1101/2023.11.17.567581)
Supplement: Supplement 1 [file media-1.pdf]

Supplementary information

Deep learning and direct sequencing of labeled RNA captures transcriptome dynamics

Vlastimil Martinek[1, 2, 3, *], Jessica Martin[1, 4, *], Cedric Belair[1], Matthew J Payea[1], Sulochan Malla[1], Panagiotis Alexiou[5, $], Manolis Maragkakis[1, $, ^].

[1] Laboratory of Genetics and Genomics, National Institute on Aging, Intramural Research Program, National Institutes of Health, Baltimore, MD 21224, USA
[2] Central European Institute of Technology, Masaryk University, 625 00 Brno, Czech Republic
[3] National Centre for Biomolecular Research, Faculty of Science, Masaryk University, 625 00 Brno, Czech Republic
[4] Center for Alzheimer's and Related Dementias, National Institute on Aging and National Institute of Neurological Disorders and Stroke, National Institutes of Health, Bethesda, MD, USA
[5] Centre for Molecular Medicine & Biobanking, University of Malta, MSD 2080 Msida, Malta

* equal contribution
$ senior author
^ contact: maragkakis@nih.gov

Dataset	Use case in this work	Generated in	Cell line	Organism
In-house HeLa	Positive and negative reads for training, validation, and testing	This work	HeLa	Homo Sapiens
In-house Neurons	Negative reads for training	This work	iPSC-derived neurons	Homo Sapiens
In-house HEK293T	Negative reads for training	This work	HEK293T	Homo Sapiens
In-house 3T3 5EU labeled reads	Testing if model captures RNA decay rates in 3T3 cells	This work	3T3	Mus Musculus
In-house HeLa 2h 5EU	Testing if model captures rna decay rates in HeLa cells	This work	HeLa	Homo Sapiens
Maier K562 classification	Positive (24 hr labeled) reads for testing classification	Maier et al. [9]	K562	Homo Sapiens
Maier K562 classification	Negative (non labeled) reads for testing classification	Maier et al. [9]	K562	Homo Sapiens
Maier K562 heat shock	Test if RNAkinet captures differential expression during stress	Maier et al. [9]	K562	Homo Sapiens

Supplementary Table 1: Description of dRNA-Seq datasets used in this work.

Experiment name	Number of reads
hsa_dRNA_HeLa_labeled_1	982840
hsa_dRNA_HeLa_nonlabeled_1	1878222
mmu_dRNA_3T3_labeled_1	620976
mmu_dRNA_3T3_labeled_2	1306329
hsa_dRNA_Hek293T_nonlabeled_1	1257185
hsa_dRNA_Neuron_nonlabeled_ctrl_1	517551
hsa_dRNA_Neuron_nonlabeled_TDP43KD_1	487902
hsa_dRNA_HeLa_5EU_2hr_1	1911933
hsa_dRNA_HeLa_5EU_2hr_2	1174797
hsa_dRNA_HeLa_5EU_2hr_3	1364193
20180514_1054_K562_5EU_1440_labeled_run	45461
20180514_1541_K562_5EU_1440_labeled_II_run	198289
20180516_1108_K562_5EU_1440_labeled_III_run	24821
20180327_1102_K562_5EU_0_unlabeled_run	144305
20180403_1102_K562_5EU_0_unlabeled_II_run	161941
20180403_1208_K562_5EU_0_unlabeled_III_run	109778

Supplementary Table 2: Raw read counts per dataset

Supplementary Figure 1

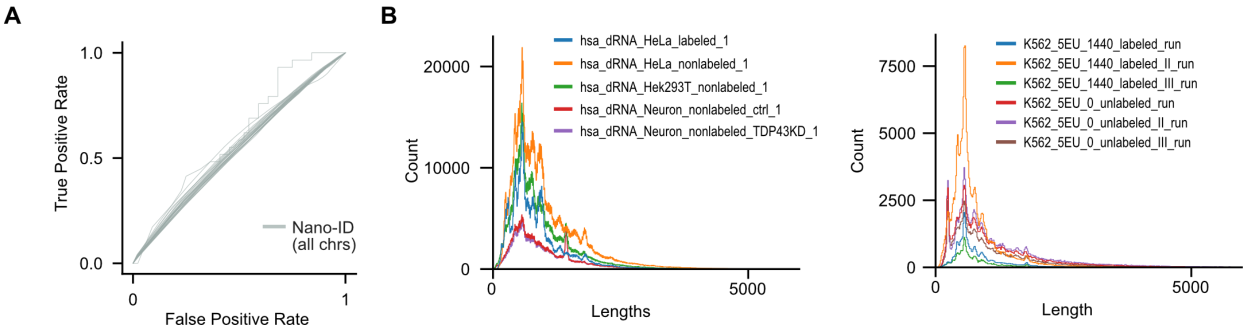

Supplementary Figure 1: A) ROC plot of the Nano-ID published in [9] on HeLa cells labeled with 5EU for 24 h. Reads are stratified by chromosome. **B**) Read length distribution for libraries used in training and testing.

Supplementary Figure 2

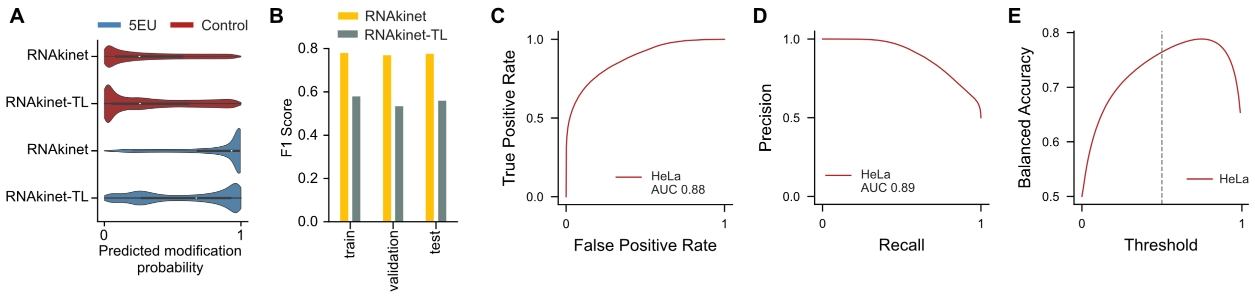

Supplementary Figure 2: A) Predicted probability distribution for positive and negative samples used in training for RNAkinet and RNAkinet-TL. **B)** Bar plot of F1 score on train, test, and validation data for RNAkinet and RNAkinet-TL. **C-E)** ROC (C), PR (D) and BA (E) plot for training data for RNAkinet.

Supplementary Figure 3

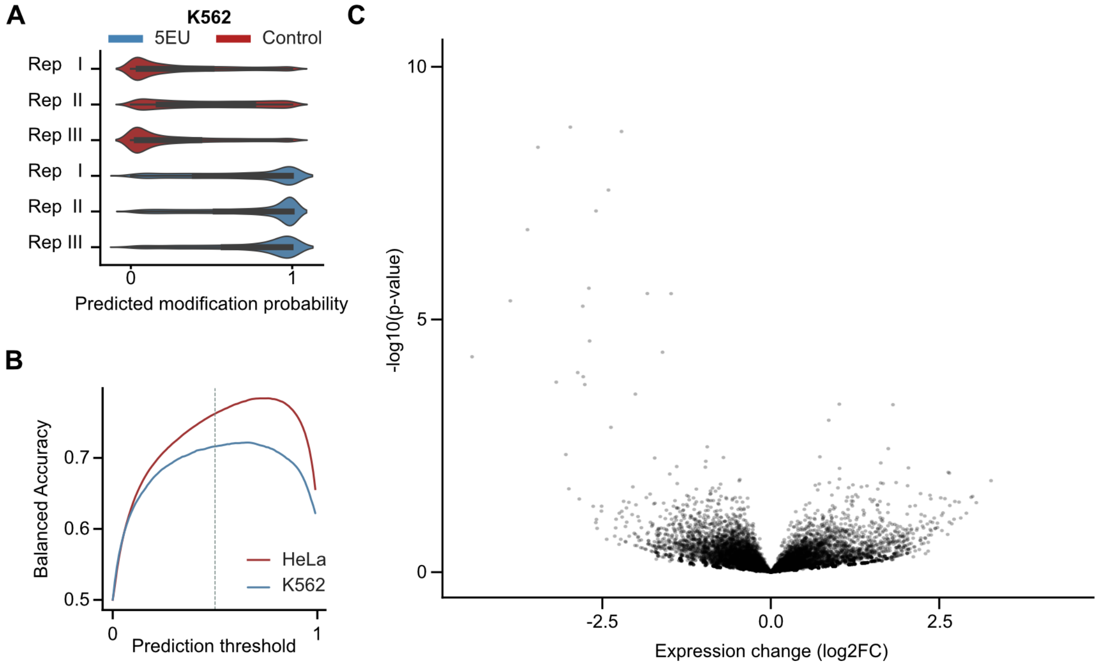

Supplementary Figure 3: A) Predicted probability distribution on reads from chromosome 1 for K562 cells **B)** BA on reads from chromosome 1 of HeLa and K562 cells. Data for HeLa cells are the same as Fig. 2 and are only included here for comparison. The threshold used for inference is marked with a gray line. **C)** Volcano plot of isoform differential expression for heat shock against control cells.

Supplementary Figure 4

A

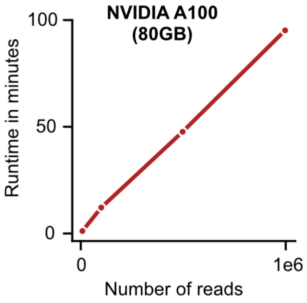

Supplementary Figure 4: A) Scatter plot of RNAkinet runtime for inference and number of reads processed.
